# (*Z*)-5-(4-Bromo­phen­yl)-3-{[(3,5-di­chloro­phen­yl)amino]­methyl­idene}furan-2(3*H*)-one

**DOI:** 10.1107/S2414314620009372

**Published:** 2020-07-17

**Authors:** Oksana A. Mayorova, Vyacheslav S. Grinev, Alevtina Yu. Yegorova

**Affiliations:** a Institute of Biochemistry and Physiology of Plants and Microorganisms, Russian Academy of Sciences, 13 Prospekt Entuziastov, Saratov 410049, Russian Federation; b Institute of Chemistry, N. G. Chernyshevsky National Research Saratov State University, Ulitsa Astrakhanskaya, 83, Saratov 410012, Russian Federation; Katholieke Universiteit Leuven, Belgium

**Keywords:** crystal structure, push–pull enamine, aryl­amino­methyl­ene derivative, furan-2(3*H*)-one, halogen substituted, π–π stacking inter­actions

## Abstract

The title compound, C_17_H_10_BrCl_2_NO_2_, crystallizes in the monoclinic space group *C*2/*c* with a large cell volume of 6207 (3) Å^3^. The asymmetric unit of the title compound investigated at 120 K contains two crystallographically independent mol­ecules (*Z*′ = 2). Each mol­ecule demonstrates slight non-planarity in the solid state and a *Z*-configuration for the exocyclic C=C bond. The crystal packing reveals the presence of π-π stacking inter­actions between the substituted benzene rings [centroid–centroid distances of 3.836 (5) Å, shift distances in the range 1.272–1.843 Å].

## Abstract 2

The mol­ecules of the push–pull enamine (*Z*)-5-(4-bromo­phen­yl)-3-{[(3,5-di­chloro­phen­yl)amino]­methyl­ene}furan-2(3*H*)-one, C_17_H_10_BrCl_2_NO_2_, are slightly non-planar in the solid state, crystallize in the *Z*-form and are involved in π–π stacking inter­actions.

## Graphical abstract

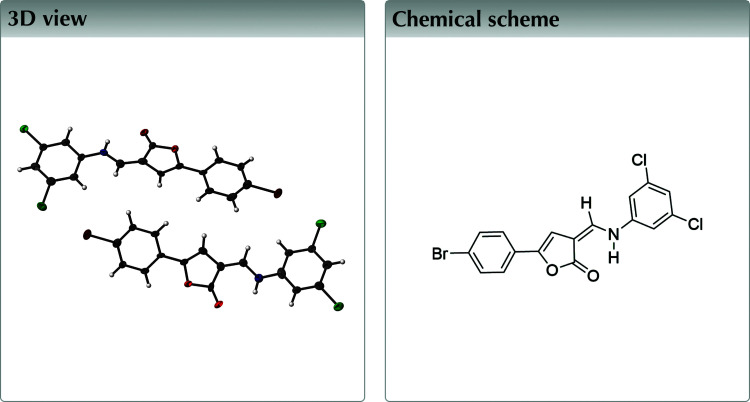

## Structure description

**Figure 1:**
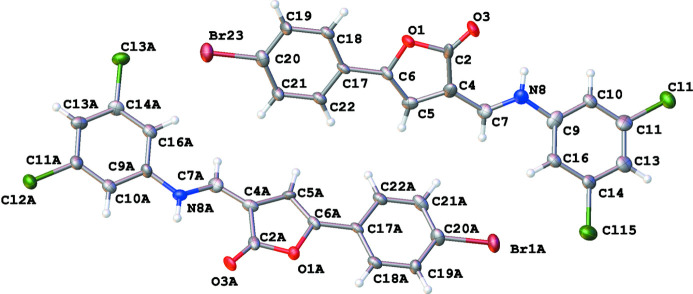
The asymmetric unit of the title compound with the atom labelling and displacement ellipsoids drawn at the 50% probability level (two crystallographically independent mol­ecules are shown).

Push–pull enamines based on furan-2(3*H*)-ones may be of inter­est for the creation of mol­ecular switches (Osipov *et al.*, 2017 ▸). Both crystallographically independent mol­ecules of the title compound are close to planarity and may be aligned together with an r.m.s.d. of 0.297 Å without and 0.561 Å with inversion. Usually, pronounced non-planar mol­ecules differ much more in their alignment with and without inversion. Actually, both mol­ecules are slightly non-planar (Fig. 1 ▸) with the 4-bromo­phenyl substituent rotated about the mean plane of the furan­one ring by approximately 2–5° [C18—C17—C6—O1 = 2.1 (12)° while the corresponding C18*A*—C17*A*—C6*A*—O1*A* torsion angle = 5.2 (13)°] . The C4=C7 as well as corresponding C4*A*=C7*A* bonds adopt a *Z* configuration. The benzene ring of the 3,5-di­chloro­phenyl substituent is also out of the plane of the mol­ecule [the dihedral angles between the mean planes of the furan­one and 3,5-di­chloro­phenyl rings are 33.6 (4) and 14.8 (4)°, respectively, for the two mol­ecules], which is a consequence of the repulsion of hydrogen atoms H16/H16*A* of the aromatic substituent and H7/H7*A* of the enamine fragment with distances H7⋯H16 = 2.178 Å and H7*A*⋯H16*A* = 2.063 Å, which is less than the sum of the van der Waals radii (2.38 Å). This is in agreement with the observation that the inter­atomic distance is slightly larger in the more twisted mol­ecule than in the more planar one.

**Figure 2:**
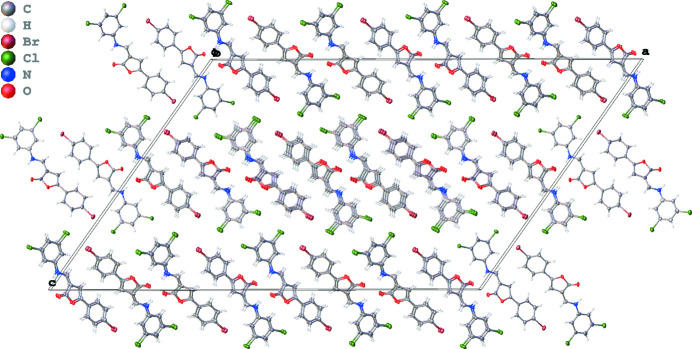
The crystal packing of the title compound, viewed along the *b* axis.

In contrast to (*Z*)-3-[(3,5-di­chloro­anilino)methyl­idene]-5-(*p*-tol­yl)furan-2(3*H*)-one (Grinev *et al.*, 2018 ▸), which demonstrated only intra­molecular hydrogen bond, in crystal of the title mol­ecule there are not only intra­molecular, but also inter­molecular hydrogen bonds (Table 1 ▸, Fig. 2 ▸). They are relatively weak but result in dimer formation in the crystal packing. The H8⋯O3 distance in both mol­ecules is significantly longer than the corresponding distance in the *p*-tolyl-substituted analogue [2.18 (2) Å]. This may be explained by the presence of the bulky electronegative bromine atom as a substituent on the benzene ring instead of a methyl group. The two mol­ecules in the asymmetric unit are oriented in a head-to-tail fashion, the bromine atom of one mol­ecule becoming relatively close to the H atoms of CH fragments of both the aromatic and enamine moieties in the neighbouring second crystallographically independent mol­ecule [H⋯Br inter­atomic distances are in the range 3.26—3.82 Å].

**Figure 3:**
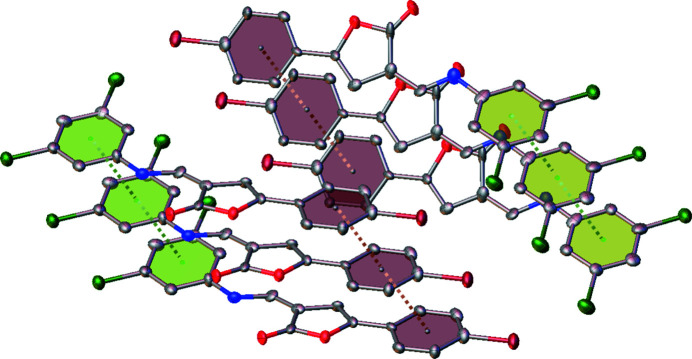
π–π stacking inter­actions between the di­chloro (green) and bromo (brown) substituted aromatic rings of the title compound.

The inter­planar distances between identically oriented mol­ecules in the *p*-tolyl substituted analogue are larger than 7 Å, excluding non-covalent inter­actions such as π–π stacking. In contrast to this, in the crystal of the title mol­ecule parallel-displaced π–π stacking inter­actions are present for both crystallographically independent mol­ecules (Fig. 3 ▸). The inter­centroid distances between the 3,5-di­chloro­phenyl as well as the 4-bromo­phenyl rings are 3.836 (5) Å for both mol­ecules, with shift distances of 1.272 and 1.665 Å for the first mol­ecule and 1.539 and 1.843 Å for the second mol­ecule.

## Synthesis and crystallization

The synthesis of the title compound was performed according to the method described by Osipov *et al.* (2017 ▸) and Grinev *et al.* (2018 ▸). Briefly, about 7 ml of benzene, 1.78 g of (12.02 mmol) triethyl orthoformate, 0.40 g (1.67 mmol) of 5-(4-bromo­phen­yl)furan-2(3*H*)-one and 0.27 g (1.67 mmol) of 3,5-di­chloro­aniline were placed into a round-bottom flask equipped with a Liebig reflux condenser, and the reaction mixture was refluxed for 2 h. The precipitate of 3-[(3,5-di­chloro­anilino)methyl­idene]-5-(4-bromo­phen­yl)furan-2(3*H*)-one was filtered off, washed with benzene and then with chloro­form, dried, and recrystallized from DMF. Yield 0.51 g (75%), yellow crystals. A single crystal suitable for X-ray analysis was obtained by slow cooling of a saturated solution of the title compound in benzene.

## Refinement

Crystal data, data collection and structure refinement details are summarized in Table 2 ▸. The relatively high *R*_int_ and *R* values as well as the low C—C bond precision are due to poor crystal quality because of probable twinning or clustering.

## Supplementary Material

Crystal structure: contains datablock(s) I. DOI: 10.1107/S2414314620009372/vm4045sup1.cif

Structure factors: contains datablock(s) I. DOI: 10.1107/S2414314620009372/vm4045Isup2.hkl

Supporting information file. DOI: 10.1107/S2414314620009372/vm4045Isup3.cml

CCDC reference: 2015222

Additional supporting information:  crystallographic information; 3D view; checkCIF report

## Figures and Tables

**Table 1:** Hydrogen-bond geometry (Å, °)

*D*—H⋯*A*	*D*—H	H⋯*A*	*D*⋯*A*	*D*—H⋯*A*
N8—H8⋯O3	0.87 (13)	2.49 (11)	3.013 (11)	119 (10)
N8—H8⋯O3^i^	0.87 (13)	2.26 (13)	3.059 (15)	153 (11)
N8*A*—H8*A*⋯O3*A*	0.88	2.44	3.050 (11)	126
N8*A*—H8*A*⋯O3*A* ^ii^	0.88	2.32	3.142 (12)	156

Symmetry codes: (i) 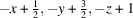 ; (ii) 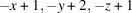 .

**Table 2:** Experimental details

Crystal data	
Chemical formula	C_17_H_10_BrCl_2_NO_2_
*M*_r_	411.07
Crystal system, space group	Monoclinic, *C*2/*c*
Temperature (K)	120
*a*, *b*, *c* (Å)	55.051 (17), 3.8355 (11), 35.979 (12)
β (°)	125.214 (6)
*V* (Å^3^)	6207 (3)
*Z*	16
Radiation type	Mo *K*α
μ (mm^−1^)	3.00
Crystal size (mm)	0.6 × 0.1 × 0.1

Data collection	
Diffractometer	Bruker APEXII CCD
Absorption correction	Multi-scan (*SADABS*; Bruker, 2014 ▸)
*T*_min_, *T*_max_	0.170, 0.337
No. of measured, independent and observed [*I* > 2σ(*I*)] reflections	17633, 6123, 3647
*R*_int_	0.136
(sin θ/λ)_max_ (Å^−1^)	0.617

Refinement	
*R*[*F* ^2^ > 2σ(*F* ^2^)], *wR*(*F* ^2^), *S*	0.092, 0.220, 1.06
No. of reflections	6123
No. of parameters	359
H-atom treatment	H atoms treated by a mixture of independent and constrained refinement
Δρ_max_, Δρ_min_ (e Å^−3^)	1.17, −1.30

Computer programs: *APEX2* (Bruker, 2013 ▸), *SAINT* (Bruker, 2013 ▸), *SHELXT* (Sheldrick, 2015*b* ▸), *SHELXL* (Sheldrick, 2015*a* ▸), *OLEX2* (Dolomanov *et al.*, 2009 ▸).

## full crystallographic data

### Crystal data

**Table:**

C_17_H_10_BrCl_2_NO_2_	*F*(000) = 3264
*M**_r_* = 411.07	*D*_x_ = 1.760 Mg m^−^^3^
Monoclinic, *C*2/*c*	Mo *K*α radiation, λ = 0.71073 Å
*a* = 55.051 (17) Å	Cell parameters from 1746 reflections
*b* = 3.8355 (11) Å	θ = 2.8–30.7°
*c* = 35.979 (12) Å	µ = 3.00 mm^−^^1^
β = 125.214 (6)°	*T* = 120 K
*V* = 6207 (3) Å^3^	Needle, metallic orangish yellow
*Z* = 16	0.6 × 0.1 × 0.1 mm

### Data collection

**Table:**

Bruker APEXII CCD diffractometer	3647 reflections with *I* > 2σ(*I*)
φ and ω scans	*R*_int_ = 0.136
Absorption correction: multi-scan (SADABS; Bruker, 2014)	θ_max_ = 26.0°, θ_min_ = 0.9°
*T*_min_ = 0.170, *T*_max_ = 0.337	*h* = −66→65
17633 measured reflections	*k* = −4→4
6123 independent reflections	*l* = −41→44

### Refinement

**Table:**

Refinement on *F*^2^	Hydrogen site location: mixed
Least-squares matrix: full	H atoms treated by a mixture of independent and constrained refinement
*R*[*F*^2^ > 2σ(*F*^2^)] = 0.092	*w* = 1/[σ^2^(*F*_o_^2^) + (0.0747*P*)^2^ + 74.2699*P*] where *P* = (*F*_o_^2^ + 2*F*_c_^2^)/3
*wR*(*F*^2^) = 0.220	(Δ/σ)_max_ = 0.001
*S* = 1.06	Δρ_max_ = 1.17 e Å^−^^3^
6123 reflections	Δρ_min_ = −1.30 e Å^−^^3^
359 parameters	Extinction correction: SHELXL2016/6 (Sheldrick 2016), Fc^*^=kFc[1+0.001xFc^2^λ^3^/sin(2θ)]^-1/4^
0 restraints	Extinction coefficient: 0.00133 (13)
Primary atom site location: dual	

### Special details

**Table:**

Geometry. All esds (except the esd in the dihedral angle between two l.s. planes) are estimated using the full covariance matrix. The cell esds are taken into account individually in the estimation of esds in distances, angles and torsion angles; correlations between esds in cell parameters are only used when they are defined by crystal symmetry. An approximate (isotropic) treatment of cell esds is used for estimating esds involving l.s. planes.
Refinement. The structure was solved by the internal phasing method and refined by the least-squares method in the anisotropic full-matrix approximation in accordance with *F*^2^*_hkl_*. Aromatic and amine H atoms refined with riding coordinates: C5(H5), C7(H7), C10(H10), C13(H13), C16(H16), C18(H18), C19(H19), C21(H21), C22(H22), N8A(H8A), C5A(H5A), C7A(H7A), C10A(H10A), C13A(H13A), C16A(H16A), C18A(H18A), C19A(H19A), C21A(H21A), C22A(H22A). Other H atoms were placed in calculated positions and refined geometrically using a riding model, with fixed thermal parameters *U*_iso_(H) = 1.2*U*_iso_(C). In addition, the following restraints and constraints were applied: *U*_anis_(C6A) = *U*_anis_(C6); *U*_anis_(C16A) = *U*_anis_(C10A) = *U*_anis_(C16) = *U*_anis_(C10); *U*_anis_(C7A) = *U*_anis_(C7); *U*_anis_(C20) = *U*_anis_(C20A); *U*_anis_(C18) = *U*_anis_(C22) = *U*_anis_(C22A) = *U*_anis_(C18A).

### Fractional atomic coordinates and isotropic or equivalent isotropic displacement parameters (Å^2^)

**Table:**

	*x*	*y*	*z*	*U*_iso_*/*U*_eq_	
Br23	0.48087 (2)	1.1512 (3)	0.68139 (4)	0.0309 (3)	
Cl1	0.14526 (6)	−0.0626 (7)	0.33686 (10)	0.0340 (7)	
Cl15	0.20333 (6)	0.2610 (8)	0.26102 (10)	0.0399 (7)	
O1	0.32912 (15)	0.9028 (18)	0.5536 (2)	0.0275 (17)	
O3	0.27944 (15)	0.865 (2)	0.5197 (2)	0.0338 (18)	
N8	0.2499 (2)	0.467 (2)	0.4316 (3)	0.029 (2)	
H8	0.247 (2)	0.48 (3)	0.453 (4)	0.035*	
C2	0.3001 (2)	0.796 (3)	0.5180 (3)	0.029 (3)	
C4	0.3023 (2)	0.614 (3)	0.4849 (3)	0.026 (2)	
C5	0.3334 (2)	0.613 (2)	0.5017 (3)	0.022 (2)	
H5	0.341698	0.512110	0.487258	0.027*	
C6	0.3483 (2)	0.784 (2)	0.5422 (3)	0.0199 (15)	
C7	0.2781 (2)	0.477 (2)	0.4441 (3)	0.0236 (16)	
H7	0.281853	0.381941	0.423527	0.028*	
C9	0.2261 (2)	0.345 (2)	0.3887 (3)	0.027 (2)	
C10	0.2008 (2)	0.216 (2)	0.3845 (3)	0.0223 (11)	
H10	0.200137	0.208946	0.410275	0.027*	
C11	0.1767 (2)	0.097 (3)	0.3425 (4)	0.030 (3)	
C13	0.1772 (2)	0.103 (2)	0.3038 (4)	0.027 (2)	
H13	0.160948	0.017098	0.275366	0.033*	
C14	0.2023 (2)	0.237 (3)	0.3086 (3)	0.023 (2)	
C16	0.2270 (2)	0.363 (2)	0.3509 (3)	0.0223 (11)	
H16	0.243784	0.458461	0.353446	0.027*	
C17	0.3802 (2)	0.867 (2)	0.5759 (3)	0.0177 (14)	
C18	0.3893 (2)	1.041 (2)	0.6163 (3)	0.0216 (11)	
H18	0.375138	1.102737	0.622215	0.026*	
C19	0.4198 (2)	1.123 (3)	0.6482 (3)	0.026 (2)	
H19	0.426177	1.240856	0.675631	0.031*	
C20	0.4401 (2)	1.031 (3)	0.6389 (4)	0.0270 (17)	
C21	0.4313 (2)	0.856 (2)	0.5991 (3)	0.022 (2)	
H21	0.445521	0.793918	0.593232	0.026*	
C22	0.4007 (2)	0.771 (2)	0.5672 (3)	0.0216 (11)	
H22	0.394359	0.647951	0.540107	0.026*	
Br1A	0.28136 (2)	−0.0323 (3)	0.32665 (4)	0.0321 (3)	
Cl2A	0.62033 (6)	1.0959 (7)	0.68860 (9)	0.0291 (6)	
Cl3A	0.55514 (6)	0.6764 (7)	0.74909 (9)	0.0316 (6)	
O1A	0.42611 (14)	0.5740 (16)	0.4455 (2)	0.0212 (15)	
O3A	0.47142 (15)	0.8240 (17)	0.4752 (2)	0.0252 (16)	
N8A	0.51085 (17)	0.717 (2)	0.5780 (3)	0.0213 (19)	
H8A	0.511802	0.804501	0.556191	0.026*	
C2A	0.4552 (2)	0.657 (2)	0.4813 (3)	0.020 (2)	
C4A	0.4599 (2)	0.516 (2)	0.5227 (3)	0.020 (2)	
C5A	0.4325 (2)	0.348 (2)	0.5088 (3)	0.019 (2)	
H5A	0.428873	0.227865	0.528260	0.023*	
C6A	0.4130 (2)	0.391 (2)	0.4639 (3)	0.0199 (15)	
C7A	0.4858 (2)	0.543 (2)	0.5660 (3)	0.0236 (16)	
H7A	0.486133	0.428201	0.589717	0.028*	
C9A	0.5354 (2)	0.766 (3)	0.6235 (3)	0.021 (2)	
C10A	0.5626 (2)	0.887 (2)	0.6326 (3)	0.0223 (11)	
H10A	0.564462	0.929666	0.608312	0.027*	
C11A	0.5865 (2)	0.944 (3)	0.6769 (3)	0.023 (2)	
C13A	0.5849 (2)	0.882 (3)	0.7136 (3)	0.026 (2)	
H13A	0.601535	0.923528	0.743953	0.031*	
C14A	0.5582 (2)	0.758 (2)	0.7044 (3)	0.023 (2)	
C16A	0.5334 (2)	0.706 (2)	0.6597 (3)	0.0223 (11)	
H16A	0.515162	0.630053	0.654176	0.027*	
C17A	0.3814 (2)	0.289 (2)	0.4306 (3)	0.0177 (14)	
C18A	0.3672 (2)	0.348 (2)	0.3842 (3)	0.0216 (11)	
H18A	0.377945	0.448435	0.373662	0.026*	
C19A	0.3374 (2)	0.260 (2)	0.3532 (3)	0.022 (2)	
H19A	0.327384	0.310506	0.321743	0.026*	
C20A	0.3224 (2)	0.097 (3)	0.3692 (4)	0.0270 (17)	
C21A	0.3361 (2)	0.038 (2)	0.4148 (4)	0.027 (2)	
H21A	0.325416	−0.068515	0.425210	0.032*	
C22A	0.3657 (2)	0.134 (2)	0.4456 (3)	0.0216 (11)	
H22A	0.375192	0.094617	0.477202	0.026*	

### Atomic displacement parameters (Å^2^)

**Table:**

	*U*^11^	*U*^22^	*U*^33^	*U*^12^	*U*^13^	*U*^23^
Br23	0.0239 (6)	0.0206 (6)	0.0413 (7)	−0.0040 (4)	0.0149 (5)	−0.0004 (5)
Cl1	0.0339 (15)	0.0288 (15)	0.0475 (17)	−0.0050 (12)	0.0282 (14)	−0.0047 (12)
Cl15	0.0389 (16)	0.0507 (19)	0.0385 (17)	0.0045 (14)	0.0273 (14)	0.0024 (14)
O1	0.025 (4)	0.036 (4)	0.030 (4)	−0.010 (3)	0.021 (3)	−0.007 (3)
O3	0.020 (4)	0.047 (5)	0.040 (5)	−0.004 (3)	0.021 (4)	−0.005 (4)
N8	0.030 (5)	0.031 (5)	0.027 (5)	−0.004 (4)	0.017 (4)	0.005 (4)
C2	0.018 (5)	0.041 (7)	0.020 (5)	−0.014 (5)	0.007 (4)	0.007 (5)
C4	0.029 (6)	0.026 (6)	0.022 (5)	−0.007 (5)	0.014 (5)	0.006 (4)
C5	0.037 (6)	0.013 (5)	0.026 (6)	−0.004 (4)	0.022 (5)	0.001 (4)
C6	0.023 (4)	0.011 (3)	0.033 (4)	−0.004 (3)	0.021 (3)	−0.003 (3)
C7	0.032 (4)	0.009 (4)	0.032 (4)	−0.004 (3)	0.020 (3)	−0.004 (3)
C9	0.033 (6)	0.008 (5)	0.028 (6)	0.005 (4)	0.010 (5)	0.003 (4)
C10	0.026 (3)	0.013 (3)	0.032 (3)	0.002 (2)	0.020 (2)	0.001 (2)
C11	0.035 (6)	0.022 (6)	0.037 (6)	0.007 (5)	0.022 (5)	−0.004 (5)
C13	0.029 (6)	0.017 (5)	0.038 (6)	0.010 (4)	0.020 (5)	0.005 (5)
C14	0.022 (5)	0.019 (5)	0.027 (6)	0.007 (4)	0.014 (5)	0.005 (4)
C16	0.026 (3)	0.013 (3)	0.032 (3)	0.002 (2)	0.020 (2)	0.001 (2)
C17	0.023 (3)	0.012 (3)	0.028 (4)	−0.001 (3)	0.021 (3)	−0.001 (3)
C18	0.027 (3)	0.017 (3)	0.030 (3)	−0.001 (2)	0.022 (2)	0.000 (2)
C19	0.023 (5)	0.028 (6)	0.030 (6)	−0.012 (4)	0.018 (5)	−0.008 (5)
C20	0.027 (4)	0.018 (4)	0.037 (4)	−0.003 (3)	0.019 (3)	0.004 (3)
C21	0.021 (5)	0.017 (5)	0.030 (6)	−0.004 (4)	0.017 (5)	−0.006 (4)
C22	0.027 (3)	0.017 (3)	0.030 (3)	−0.001 (2)	0.022 (2)	0.000 (2)
Br1A	0.0217 (5)	0.0293 (7)	0.0428 (7)	−0.0055 (5)	0.0171 (5)	−0.0041 (5)
Cl2A	0.0262 (13)	0.0328 (15)	0.0328 (14)	−0.0045 (11)	0.0196 (12)	−0.0021 (12)
Cl3A	0.0424 (16)	0.0316 (15)	0.0262 (14)	−0.0033 (12)	0.0228 (13)	0.0014 (11)
O1A	0.020 (3)	0.019 (4)	0.024 (4)	−0.004 (3)	0.013 (3)	−0.001 (3)
O3A	0.025 (4)	0.024 (4)	0.036 (4)	−0.011 (3)	0.023 (3)	−0.004 (3)
N8A	0.028 (5)	0.020 (5)	0.021 (4)	−0.001 (4)	0.017 (4)	0.000 (3)
C2A	0.019 (5)	0.015 (5)	0.030 (6)	−0.003 (4)	0.015 (4)	−0.009 (4)
C4A	0.034 (6)	0.008 (5)	0.031 (6)	0.001 (4)	0.025 (5)	0.002 (4)
C5A	0.027 (5)	0.020 (5)	0.016 (5)	−0.003 (4)	0.016 (4)	0.000 (4)
C6A	0.023 (4)	0.011 (3)	0.033 (4)	−0.004 (3)	0.021 (3)	−0.003 (3)
C7A	0.032 (4)	0.009 (4)	0.032 (4)	−0.004 (3)	0.020 (3)	−0.004 (3)
C9A	0.018 (5)	0.022 (6)	0.025 (5)	−0.003 (4)	0.013 (4)	0.002 (4)
C10A	0.026 (3)	0.013 (3)	0.032 (3)	0.002 (2)	0.020 (2)	0.001 (2)
C11A	0.022 (5)	0.021 (6)	0.029 (6)	0.002 (4)	0.016 (5)	−0.002 (4)
C13A	0.033 (6)	0.023 (6)	0.026 (6)	0.009 (5)	0.019 (5)	0.006 (4)
C14A	0.034 (6)	0.008 (5)	0.031 (6)	0.002 (4)	0.021 (5)	0.006 (4)
C16A	0.026 (3)	0.013 (3)	0.032 (3)	0.002 (2)	0.020 (2)	0.001 (2)
C17A	0.023 (3)	0.012 (3)	0.028 (4)	−0.001 (3)	0.021 (3)	−0.001 (3)
C18A	0.027 (3)	0.017 (3)	0.030 (3)	−0.001 (2)	0.022 (2)	0.000 (2)
C19A	0.019 (5)	0.020 (5)	0.024 (5)	−0.002 (4)	0.011 (4)	0.002 (4)
C20A	0.027 (4)	0.018 (4)	0.037 (4)	−0.003 (3)	0.019 (3)	0.004 (3)
C21A	0.032 (6)	0.014 (5)	0.046 (7)	0.002 (4)	0.029 (6)	0.006 (5)
C22A	0.027 (3)	0.017 (3)	0.030 (3)	−0.001 (2)	0.022 (2)	0.000 (2)

### Geometric parameters (Å, º)

**Table:**

Br23—C20	1.907 (10)	Br1A—C20A	1.926 (10)
Cl1—C11	1.732 (11)	Cl2A—C11A	1.756 (10)
Cl15—C14	1.746 (10)	Cl3A—C14A	1.740 (10)
O1—C2	1.412 (11)	O1A—C2A	1.395 (11)
O1—C6	1.409 (11)	O1A—C6A	1.416 (11)
O3—C2	1.204 (12)	O3A—C2A	1.220 (11)
N8—H8	0.86 (10)	N8A—H8A	0.8800
N8—C7	1.342 (13)	N8A—C7A	1.361 (12)
N8—C9	1.411 (13)	N8A—C9A	1.412 (12)
C2—C4	1.445 (15)	C2A—C4A	1.457 (13)
C4—C5	1.448 (14)	C4A—C5A	1.440 (13)
C4—C7	1.397 (14)	C4A—C7A	1.382 (14)
C5—H5	0.9500	C5A—H5A	0.9500
C5—C6	1.360 (13)	C5A—C6A	1.339 (13)
C6—C17	1.486 (13)	C6A—C17A	1.487 (13)
C7—H7	0.9500	C7A—H7A	0.9500
C9—C10	1.400 (14)	C9A—C10A	1.417 (13)
C9—C16	1.389 (14)	C9A—C16A	1.388 (13)
C10—H10	0.9500	C10A—H10A	0.9500
C10—C11	1.393 (14)	C10A—C11A	1.378 (14)
C11—C13	1.410 (15)	C11A—C13A	1.392 (14)
C13—H13	0.9500	C13A—H13A	0.9500
C13—C14	1.390 (14)	C13A—C14A	1.390 (14)
C14—C16	1.418 (14)	C14A—C16A	1.400 (14)
C16—H16	0.9500	C16A—H16A	0.9500
C17—C18	1.403 (13)	C17A—C18A	1.392 (13)
C17—C22	1.380 (12)	C17A—C22A	1.391 (13)
C18—H18	0.9500	C18A—H18A	0.9500
C18—C19	1.417 (13)	C18A—C19A	1.394 (13)
C19—H19	0.9500	C19A—H19A	0.9500
C19—C20	1.385 (14)	C19A—C20A	1.393 (14)
C20—C21	1.391 (14)	C20A—C21A	1.375 (14)
C21—H21	0.9500	C21A—H21A	0.9500
C21—C22	1.427 (13)	C21A—C22A	1.392 (14)
C22—H22	0.9500	C22A—H22A	0.9500
			
C6—O1—C2	106.7 (8)	C2A—O1A—C6A	107.7 (7)
C7—N8—H8	118 (8)	C7A—N8A—H8A	118.4
C7—N8—C9	122.8 (9)	C7A—N8A—C9A	123.3 (8)
C9—N8—H8	117 (8)	C9A—N8A—H8A	118.4
O1—C2—C4	107.4 (9)	O1A—C2A—C4A	107.1 (8)
O3—C2—O1	119.9 (10)	O3A—C2A—O1A	121.4 (9)
O3—C2—C4	132.6 (9)	O3A—C2A—C4A	131.5 (9)
C2—C4—C5	107.3 (9)	C5A—C4A—C2A	106.1 (8)
C7—C4—C2	124.4 (10)	C7A—C4A—C2A	125.8 (9)
C7—C4—C5	128.3 (10)	C7A—C4A—C5A	128.1 (9)
C4—C5—H5	126.8	C4A—C5A—H5A	125.8
C6—C5—C4	106.4 (9)	C6A—C5A—C4A	108.3 (8)
C6—C5—H5	126.8	C6A—C5A—H5A	125.8
O1—C6—C17	115.2 (8)	O1A—C6A—C17A	115.4 (8)
C5—C6—O1	112.2 (8)	C5A—C6A—O1A	110.8 (8)
C5—C6—C17	132.5 (9)	C5A—C6A—C17A	133.8 (9)
N8—C7—C4	125.5 (10)	N8A—C7A—C4A	126.2 (9)
N8—C7—H7	117.2	N8A—C7A—H7A	116.9
C4—C7—H7	117.2	C4A—C7A—H7A	116.9
C10—C9—N8	118.4 (9)	N8A—C9A—C10A	119.3 (8)
C16—C9—N8	121.2 (10)	C16A—C9A—N8A	121.8 (9)
C16—C9—C10	120.3 (9)	C16A—C9A—C10A	118.9 (9)
C9—C10—H10	120.0	C9A—C10A—H10A	120.2
C11—C10—C9	120.0 (10)	C11A—C10A—C9A	119.7 (9)
C11—C10—H10	120.0	C11A—C10A—H10A	120.2
C10—C11—Cl1	120.5 (8)	C10A—C11A—Cl2A	120.0 (8)
C10—C11—C13	121.0 (10)	C10A—C11A—C13A	122.1 (10)
C13—C11—Cl1	118.6 (8)	C13A—C11A—Cl2A	117.9 (8)
C11—C13—H13	121.0	C11A—C13A—H13A	121.0
C14—C13—C11	118.1 (10)	C14A—C13A—C11A	117.9 (10)
C14—C13—H13	121.0	C14A—C13A—H13A	121.0
C13—C14—Cl15	119.3 (8)	C13A—C14A—Cl3A	119.7 (8)
C13—C14—C16	121.7 (10)	C13A—C14A—C16A	121.3 (9)
C16—C14—Cl15	119.0 (8)	C16A—C14A—Cl3A	119.0 (8)
C9—C16—C14	118.9 (9)	C9A—C16A—C14A	120.1 (9)
C9—C16—H16	120.6	C9A—C16A—H16A	119.9
C14—C16—H16	120.6	C14A—C16A—H16A	119.9
C18—C17—C6	119.6 (8)	C18A—C17A—C6A	120.7 (8)
C22—C17—C6	119.6 (8)	C22A—C17A—C6A	119.9 (9)
C22—C17—C18	120.8 (9)	C22A—C17A—C18A	119.3 (9)
C17—C18—H18	120.2	C17A—C18A—H18A	119.7
C17—C18—C19	119.5 (9)	C17A—C18A—C19A	120.5 (9)
C19—C18—H18	120.2	C19A—C18A—H18A	119.7
C18—C19—H19	120.3	C18A—C19A—H19A	120.6
C20—C19—C18	119.3 (9)	C20A—C19A—C18A	118.7 (9)
C20—C19—H19	120.3	C20A—C19A—H19A	120.6
C19—C20—Br23	119.0 (8)	C19A—C20A—Br1A	119.3 (8)
C19—C20—C21	121.5 (9)	C21A—C20A—Br1A	119.2 (8)
C21—C20—Br23	119.4 (8)	C21A—C20A—C19A	121.5 (10)
C20—C21—H21	120.5	C20A—C21A—H21A	120.4
C20—C21—C22	119.0 (9)	C20A—C21A—C22A	119.3 (9)
C22—C21—H21	120.5	C22A—C21A—H21A	120.4
C17—C22—C21	119.8 (9)	C17A—C22A—C21A	120.6 (9)
C17—C22—H22	120.1	C17A—C22A—H22A	119.7
C21—C22—H22	120.1	C21A—C22A—H22A	119.7

### Hydrogen-bond geometry (Å, º)

**Table:**

*D*—H···*A*	*D*—H	H···*A*	*D*···*A*	*D*—H···*A*
N8—H8···O3	0.87 (13)	2.49 (11)	3.013 (11)	119 (10)
N8—H8···O3^i^	0.87 (13)	2.26 (13)	3.059 (15)	153 (11)
N8*A*—H8*A*···O3*A*	0.88	2.44	3.050 (11)	126
N8*A*—H8*A*···O3*A*^ii^	0.88	2.32	3.142 (12)	156

Symmetry codes: (i) −*x*+1/2, −*y*+3/2, −*z*+1; (ii) −*x*+1, −*y*+2, −*z*+1.
